# Analysis of Proteolytic Processes and Enzymatic Activities in the Generation of Huntingtin N-Terminal Fragments in an HEK293 Cell Model

**DOI:** 10.1371/journal.pone.0050750

**Published:** 2012-12-07

**Authors:** Andrew T. N. Tebbenkamp, Keith W. Crosby, Zoe B. Siemienski, Hilda H. Brown, Todd E. Golde, David R. Borchelt

**Affiliations:** 1 Department of Neuroscience, Center for Translational Research in Neurodegenerative Disease, McKnight Brain Institute, University of Florida, Gainesville, Florida, United States of America; 2 SantaFe HealthCare Alzheimer's Disease Research Center, University of Florida, Gainesville, Florida, United States of America; Emory University, United States of America

## Abstract

**Background:**

N-terminal fragments of mutant huntingtin (htt) that terminate between residues 90–115, termed cleavage product A or 1 (cp-A/1), form intracellular and intranuclear inclusion bodies in the brains of patients with Huntington's disease (HD). These fragments appear to be proteolytic products of the full-length protein. Here, we use an HEK293 cell culture model to investigate huntingtin proteolytic processing; previous studies of these cells have demonstrated cleavage of htt to cp-A/1 like htt fragments.

**Results:**

Recombinant N-terminal htt fragments, terminating at residue 171 (also referred to as cp-B/2 like), were efficiently cleaved to produce cp-A/1 whereas fragments representing endogenous caspase, calpain, and metalloproteinase cleavage products, terminating between residues 400–600, were inefficiently cleaved. Using cysteine-labeling techniques and antibody binding mapping, we localized the C-terminus of the cp-A/1 fragments produced by HEK293 cells to sequences minimally limited by cysteine 105 and an antibody epitope composed of residues 115–124. A combination of genetic and pharmacologic approaches to inhibit potential proteases, including γ-secretase and calpain, proved ineffective in preventing production of cp-A/1.

**Conclusions:**

Our findings indicate that HEK293 cells express a protease that is capable of efficiently cleaving cp-B/2 like fragments of htt with normal or expanded glutamine repeats. For reasons that remain unclear, this protease cleaves longer htt fragments, with normal or expanded glutamine expansions, much less efficiently. The protease in HEK293 cells that is capable of generating a cp-A/1 like htt fragment may be a novel protease with a high preference for a cp-B/2-like htt fragment as substrate.

## Introduction

Huntington's disease (HD) is an autosomal-dominant, progressive, and fatal neurodegenerative disease that is caused by an expansion of a CAG trinucleotide repeat in the first exon of the *htt* gene (GenBank:NM_002111) [1]. Disease invariably occurs when the CAG repeat in the first exon, which codes for glutamine (Q), expands to ≥39 consecutive CAG repeats. Patients carrying the polyQ expansion exhibit general brain atrophy, prominent cortical- and striatal-neuron loss, and harbor inclusion bodies composed of mutant htt throughout their central nervous system [2]–[4]. These inclusion bodies are found in the nucleus and cytoplasm, and react to antibodies recognizing N-terminal epitopes of htt [5], [6]. Lunkes and co-workers identified two N-terminal htt cleavage products in HD brains and in cell culture models that were termed cleavage product-A (cp-A, C-terminus mapped to amino acids 104–114 in cell models) and cleavage product-B (cp-B, C-terminus mapped to amino acids 146–214) [7]. The antibody binding characteristics of the pathologic inclusion bodies in HD brain are consistent with cp-A/1 sized fragments in that nuclear inclusions, and most cytoplasmic inclusions, are composed of a htt cleavage product whose C-terminus lies between antibody epitopes composed of residues 85–90 and 115–124 [7], [8]. These antibody binding characteristics are shared by inclusions throughout the CNS, and thus it appears that the protease responsible for generating cp-A/1 is expressed broadly in CNS neurons. More recently, a report by Landles *et al.* provided evidence that the smallest N-terminal fragment that is produced in the brains of *Hdh*Q150 knock-in mice (the presumed cp-A equivalent) may be generated by cleavage at residue 90 [9]. Thus, although there is agreement regarding the approximate length of the pathologic mutant htt fragment, the precise length of this fragment remains unclear.

Many studies suggest that shorter htt fragments are more toxic than the full-length protein. Cells transfected with vectors expressing N-terminal fragments of mutant htt show more aggregates and are more susceptible to apoptotic stimuli than cells transfected with full-length mutant htt [10], [11]. Similarly, transgenic mice that express mutant N-terminal htt fragments exhibit more robust phenotypes than their counterparts expressing full-length mutant htt. For example, the R6/2 mice and N171-82Q mice, which express the first exon of htt and the first 171 amino acids (into the fourth exon), respectively, exhibit robust HD-like inclusion pathology and die at three months and six months, respectively [12], [13]. In contrast, the YAC128 and BACHD mice express full-length htt (67 exons) but are slower to develop HD-like inclusion pathology and live much longer [14], [15]. Similarly, mice with an expanded polyQ knocked into their endogenous htt allele are also slower to exhibit HD-like inclusion pathology and live normal lifespans [for review see [16]]. A comparison between knock-in mice (*Hdh*Q150) and R6/2 mice showed that the progressive behavioral impairments and aggregate deposition of the *Hdh*Q150 mice were similar to those of the R6/2 mice, but of a slower and delayed progression [17].

The precise location of the C-terminus of cp-A-sized htt fragments is unknown but, as described above, it is thought to be within a region bounded by residues 90 and 115. Several laboratories have developed cell culture models that recapitulate proteolytic processing of htt and most information regarding the C-terminus of cp-A sized fragments has come from studies of these models. Lunkes and co-workers developed a model in rodent NG108 cells in which expression of an htt construct that included the first 502 residues of htt was cleaved to produce cp-A as well as cp-B [7]. Further, the authors showed that mutating C109 and E110 to alanines, and mutating N111 and I112 to alanines within an N502 htt construct led to decreased production of cp-A sized fragments. Another report by Kim *et al.* produced evidence that also suggested the C-terminus of cp-A lies between 104 and 114 [18]. In previous work in an HEK293 cell model in which a cp-B sized htt fragment was expressed (N171), we also narrowed the C-terminus of cp-A to between residues 90 and 115 [8]. However, in a PC12 cell model in which full-length htt was expressed, Ratovitski and co-workers found no effect on cp-A generation when they deleted amino acids 105–114 of their htt construct and concluded that the PC12 cell model generated novel htt cleavage products and termed the smallest N-terminal fragment cp-1 [19]. In the PC12 cell model, Ratovitski and colleagues also observed a cp-B sized fragment, which they termed cp-2 and further demonstrated that it encompasses residues 1 to 167 [20]. Apart from cp-A/1 and cp-B/2, there are six well-defined htt cleavage products, generated by calpains at residues 469 and 536, caspases at residues 513, 552, and 586, or matrix metalloproteinase-10 (MMP-10) at residue 402 [21]–[24].

In this current report, we have further evaluated whether HEK293 cells are useful models to examine htt proteolytic cleavage. We utilized bioinformatic and gene expression analysis, pharmacological inhibition, and various htt substrates to further characterize this model. Our data suggest that a novel proteolytic activity is expressed by these cells that can cleave htt to produce a cp-A/1 like fragment. This fragment possesses a C-terminus of that is minimally limited by residues 105–115.

## Methods

### Reagents

Cell culture media used was DMEM/High glucose (HyClone) supplemented with 10% horse serum and 2 mM L-glutamine. For transfections, DNA and Lipofectamine were diluted in Opti-MEM following the manufacturer's protocol (Invitrogen, Carlsbad, CA). Antibodies used were: htt1-17, a generous gift from Dr. Ricky Hirschhorn (Hood College); 1H6, 2B4, and EM48 were purchased from Millipore (Boston, MA); htt81-90 was previously described [8]; htt3-16 and htt64-82 were purchased from Sigma-Aldrich (St. Louis, MO); LC3 was a generous gift from Dr. William Dunn Jr. (University of Florida) [25]; anti-activated calpain-1 was a generous gift from Dr. Kevin Wang (University of Florida, Department of Psychiatry, Gainesville, FL) [26]; anti-mouse and anti-rabbit antibodies conjugated to HRP were purchased from Kirkegaard & Perry Laboratories (Gaithersburg, MD).

### Generation of plasmids

To make N586-18Q, we used cDNA encoding htt amino acids 171–586 generated by GenScript (Piscataway, NJ). This cDNA was excised from its vector using *XhoI* and *SalI* then ligated into the vector pcDNA3.1(+) encoding htt N171-18Q [8]. Other htt lengths were generated by standard PCR techniques using the htt 171–586 cDNA as a template and primers shown in Table S1. All vectors were sequenced to verify the number of glutamines and coding sequence at the University of Florida Interdisciplinary Center for Biotechnology Research.

### Cell transfections and immunoblots

Human embryonic kidney 293 (HEK293) cells were plated in 60 mm poly-D-lysine-coated dishes 24 hours before transfection. Once cells were 80% confluent, they were transfected with Lipofectamine 2000 (Invitrogen, Carlsbad, CA) using the manufacturer's protocol. Media was exchanged 24 hours post-transfection and cells were harvested at 48 hours post-transfection. In relevant experiments, pharmacological inhibitors were added at the 24-hour time point with the media exchange. The inhibitors used are as follows (targets in parentheses): Ucf-101 (HtrA2/Omi); AEBSF at 0.1 mM (serine proteases); PMSF at 1.0 mM (serine proteases); Leupeptin at 20 µM (trypsin-like and cysteine proteases); Pepstatin A at 1 µM and 100 µM (aspartyl proteases); E-64 at 50 µM (cysteine proteases); EDTA at 5 mM (metal chelator/metallopeptidases); EGTA at 5 mM (calcium chelator); Bestatin Methyl Ester at 100 µM (aminopeptidases), MG-132 at 10 µM (proteasome); Chloroquine at 100 µM (lysosome neutralizer); 3-methyladenine at 10 mM (autophagy inhibitor); Rapamycin at 0.2 µM (mTOR, autophagy activator); MDL28170 at 30 µM (calpain-1 and calpain-2); DAPT N-[N- (3,5-difluorophenacetyl)-L-alanyl]-S-phenylglycine t-butyl ester] at 20 µM (γ-secretase); LY411,575 [N(2)-[(2S)-2-(3,5-difluorophenyl)-2-hydroxyethanoyl]-N(1)-[(7S)-5-methyl-6-oxo-6,7-dihydro-5H-dibenzo[b,d]azepin-7-yl]-L-alaninamide] at 1 µM (γ-secretase). Chemicals were purchased from Calbiochem (San Diego, CA) or Sigma-Aldrich (St. Louis, MO), except for DAPT and LY411,575, which were purchased from Cayman Chemical Co (Ann Arbor, MI) or were kindly provided by Abdul Fauq (Mayo Clinic Jacksonville, Jacksonville, FL) [27], respectively. Pharmacological inhibitors were resuspended in their appropriate diluent, aliquoted, and frozen at −80°C until use within approximately 1–2 weeks of purchase for all experiments.

Cells were collected and lysed in PBS with protease inhibitor cocktail (Sigma, P8340) by brief sonication with a probe sonicator (Microson XL2000, 2 watts at 22.5 kHz; Misonix, Farmingdale, NY), followed by a brief centrifugation at 3000× g to pellet membranous material. The protein concentration of cell lysate supernatant was determined with a colorimetric BCA assay (Pierce, Rockford, IL). Equal amounts of protein were boiled in Laemmli buffer [28] then resolved by SDS-PAGE through 4–20% Tris-Glycine gels (Invitrogen, Carlsbad, CA). Proteins were then electrophoresed to a nitrocellulose membrane (BA85, Whatman Inc., Piscataway, NJ) and incubated with primary antibodies indicated in the figure legends. Standard chemiluminescence (Pierce, Rockford, IL) was used to visualize proteins using a Fuji imaging system (FUJIFILM Life Science, Stamford, CT).

### Biotinylation of huntingtin

HEK293 cells transfected with cDNA encoding htt N171-18Q were harvested at 48 hours. Cell lysates were collected as described above. The supernatant was split into two tubes: one mixed with immunoprecipitation buffer (0.2% SDS and 0.4% deoxycholate) and subsequently immunoprecipitated with the htt64-82 antibody; the second tube was mixed with EZ-link Biotin-BMCC (Pierce, Rockford, IL) followed by immunoprecipitation with htt64-82. Each tube was then mixed with Laemmli buffer [28] and loaded onto two 4–20% Tris-Glycine gels (Invitrogen, Carlsbad, CA) to be subsequently analyzed by Streptavidin (ExtrAvidin-Peroxidase, Sigma-Aldrich, St. Louis, MO) or htt3-16 antibodies.

### Expression of huntingtin in *E. coli*

PCR was used to amplify htt N171-18Q from pcDNA3.1(+) and the amplicon was ligated into pGEX-4T-2 (GE Healthcare-Lifesciences, Piscataway, NJ). GST-N171-18Q was expressed in *E. coli* (DH5á, Invitrogen, Carlsbad, CA) and subsequently purified and cleaved from GST using Glutathione Sepharose beads (GE Healthcare-Lifesciences, Piscataway, NJ) and a Thrombin Cleavage Capture Kit (Novagen, San Diego, CA), respectively, following manufacturer's protocols.

### Stable cell shRNAi Calpain1

HEK293 cells were transfected with pGIPZ vector encoding shRNAi against human calpain-1 (Open Biosystems, Huntsville, AL, Catalog # RHS4186-97547463). Cells were selected for resistance to Puromycin (1 ug/mL, Sigma-Aldrich, St. Louis, MO) and grown to single colonies. One colony was expanded then sorted for highest GFP expression using flow cytometry.

### RNA Extraction and Microarray

Total RNA from HEK293 and mouse LTK(-) cells was extracted using the TRIzol reagent following the manufacturer's protocol (Invitrogen). cRNA was then generated from the RNA template and labeled using a one-color Quick-Amp Labeling Kit (Agilent, Product #5190-0442, Santa Clara, CA) and followed by purification using Qiagen RNeasy Kit (Qiagen, Product #74104, Valencia, CA). Microarrays for human (Agilent, GE 4 x 44K, #014850) and for mouse (Agilent, GE 4 x 44K, #014868) and labeled cRNA were given to University of Florida Interdisciplinary Center for Biotechnology Research for hybridization and microarray reading. The raw data sets for protease genes in Excel format are provided as Data File S1 and S2. Protease genes with average expression intensities higher than the average intensities for negative controls were compiled within a cell line, and then compared across cell lines to determine which proteases were specifically expressed in the HEK293 cell line.

## Results

### Substrate specificity of the cp-A/1 generating protease(s) in HEK293 cells

**Figure 1:**
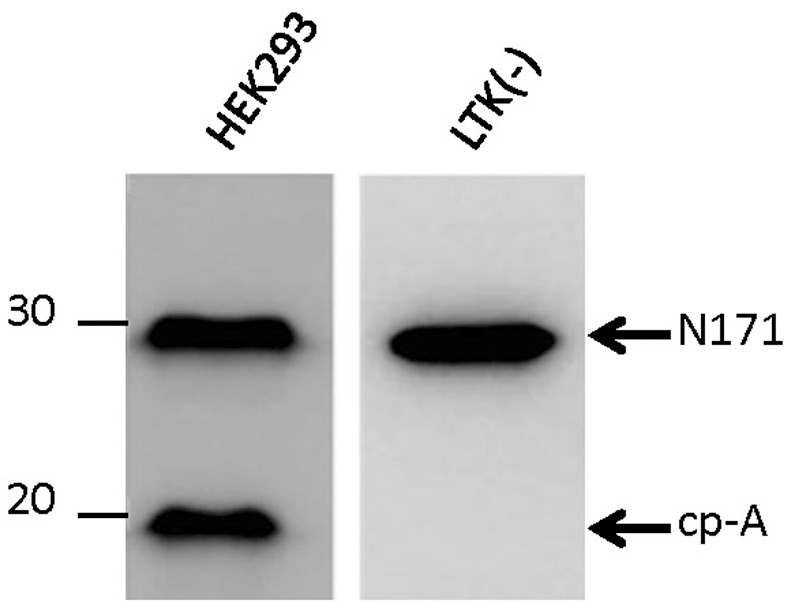
HEK293 cells but not mouse L cells cleave htt to produce a cp-A/1 like fragment. HEK293 and mouse L cells [thymidine kinase negative (TK^−^)] were transiently transfected with httN171-18Q constructs. After 48 hours, the cells were harvested and analyzed by immunoblot with the htt antibody 2B4. Only the HEK293 cells produce a cleavage product. The image shown is representative of at least 3 repetitions.

The model system we used in this study is based on expression of htt N171-18Q fragments in HEK293 cells, in which we observe the production of a cleavage product of cp-A/1 size (Fig. 1). HEK293 cells are derived from human embryonic kidney and are fibroblastic in appearance, but transcriptional profiling of these cells has demonstrated that their expression profile overlaps with neural cell types [29]. Notably, a cell line derived from mouse lung fibroblasts (LTK^−^) does not show cleavage of htt N171-18Q to a cp-A/1 sized fragment (Fig. 1).

**Figure 2:**
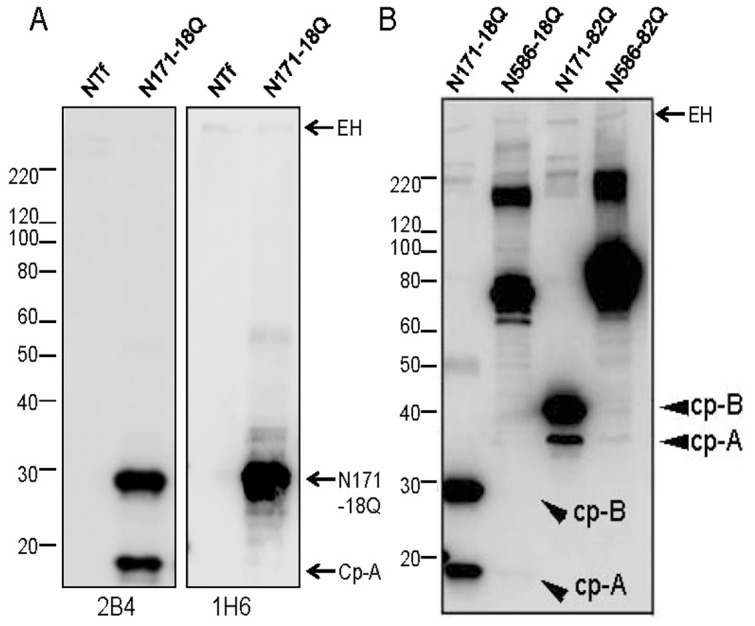
Polyglutamine length does not affect cleavage efficiency. A, HEK293 cells were non-transfected (NTf) or transfected with cDNA encoding htt N171-18Q. Cell lysates were analyzed by immunoblots incubated with antibodies 2B4 or 1H6. B, HEK293 cells were transfected with htt cDNAs shown at the top of the figure, followed by lysis and SDS-PAGE. Arrowheads identify substrates or cleavage products that are generated at approximately equally levels for short and expanded polyglutamine lengths. The lack of an effect of polyglutamine length on cleavage efficiency was observed for both N171 and N586 based constructs. Immunoblots were probed with the htt81-90 antibody (1∶3000). The images shown are representative of at least 3 repetitions of the experiment. EH = endogenous htt.

The N171-18Q cleavage product produced in HEK293 cells binds antibodies N-terminal to amino acid 115 but lacks reactivity to the 1H6 antibody, which recognizes amino acids 115–124 (Fig. 2A) [8]. To avoid issues regarding the aggregation of huntingtin fragments with expanded polyglutamine repeats, we primarily used htt constructs with normal repeat lengths. In our hands, in these cells, htt N171 substrates with normal repeat lengths were cleaved as efficiently as N171 substrates with expanded repeats (Fig. 2B). Longer substrates such as htt N586 (fragments encompassing the first 586 amino acids of htt that would be generated by caspase 6 cleavage [21], [22]) were cleaved inefficiently whether the repeat length is short (18Q) or expanded (82Q) (Fig. 2B; the arrows on panel B mark the expected positions of cp-A and cp-B like fragments derived from N586 constructs). To determine whether other htt N-terminal fragments generated by calpain, caspase, or MMP-10 are more efficient substrates for subsequent cleavage, we transfected htt cDNAs encoding 18Q versions of these htt fragments into HEK293 cells and then assessed the generation of cp-A/1 and cp-B/2 by immunoblot (Fig. S1). Htt fragments that would be generated by calpain, caspase, and MMP-10 endoproteolysis (N402, N469, N513, N536, N552, and N586) were capable of generating only small amounts of fragments that migrated at the sizes expected for cp-A/1 and cp-B/2. These data establish that N-terminal htt fragments of a size similar to natural cp-B/2 are a preferred substrate of the protease in HEK293 cells that is capable of generating a cp-A/1-like fragment.

**Figure 3:**
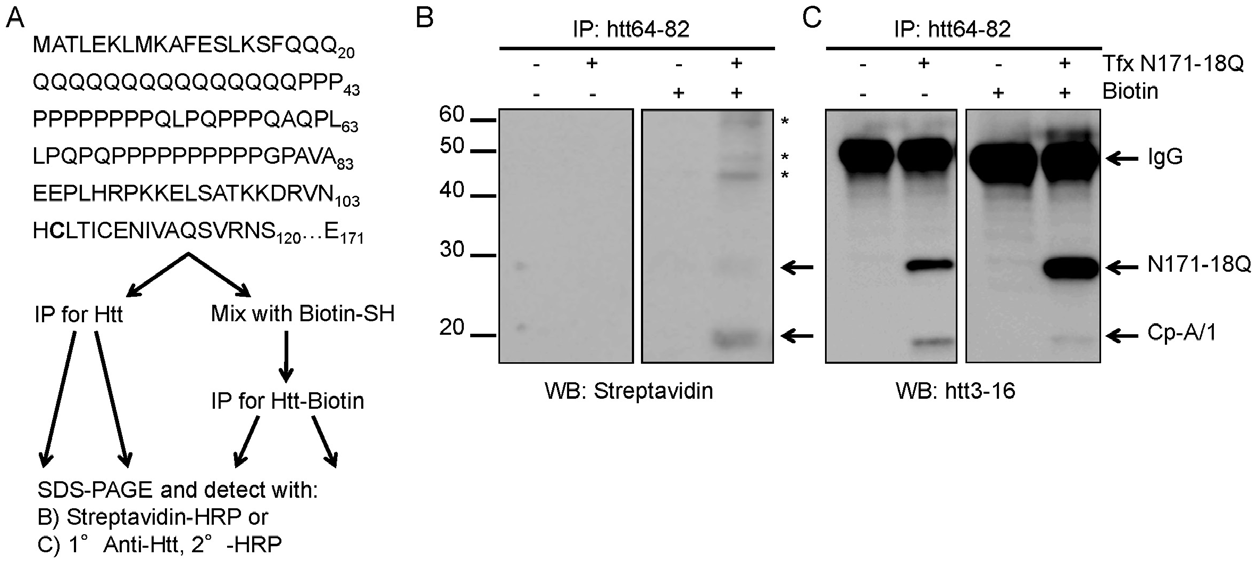
Htt cleavage occurs C-terminal to cysteine 105. A, The sequence of htt is shown through residue 171, the substrate used in these experiments. The first cysteine in htt is at residue 105 (bold) and represents the most N-terminal cysteine that could be labeled with sulfhydrylated biotin (Biotin-SH). This cysteine must be present for any labeling to occur. Subsequent immunoprecipitation (IP) followed by detection with streptavidin or htt antibodies was used to visualize the biotinylation. B, Detection using streptavidin-HRP of transfected cell lysate (Tfx N171-18Q, +) reacted with biotinylation reagent (Biotin, +) shows cp-B/2 and cp-A/1-sized products (arrows). Asterisks (*) signify possible multimers of htt. C, Detection of htt with the antibody htt3-16 confirms the identities of cp-B/2 and cp-A/1 (bottom two arrows). IgG from the immunoprecipitation is observed in all lanes (top arrow). The images shown are representative of at least 3 repetitions of the experiment.

To gain insight into the site of cleavage to generate cp-A/1, we asked whether the cp-A/1-sized fragment that is generated by proteolysis of N171-18Q (cp-B/2-like) could be covalently modified by biotinylation reagents that specifically react with cysteine residues (Fig. 3A). The N171-18Q cDNA possesses 4 cysteine resides located at 105, 109, 137, and 152. If proteolysis occurs C-terminal to residue 105 or 109, then cysteines 105 and/or 109 would be present for reactivity, allowing for detection of immunoprecipitated cp-A/1 by streptavidin conjugated to horseradish peroxidase (HRP) (Fig. 3A). In cells that had been transfected with htt N171-18Q, lysed, reacted with EZ-link Biotin BMCC, and then immunoprecipitated with htt64-82 antibody, we observed a protein band of similar size to cp-A/1 on immunoblots after detection with streptavidin HRP (Fig. 3B). This band was not found in untransfected cells, or when cell lysates were not reacted with the cysteine biotinylation reagent. In parallel, we performed an immunoblot of the htt64-82 immunoprecipitates with htt3-16 antibodies confirming efficient immunoprecipitation of htt N171-18Q and htt cp-A/1 (Fig. 3C). For reasons that remain unclear, detection of uncleaved N171-18Q (cp-B/2) with streptavidin-HRP was very poor despite indications it was efficiently immunoprecipitated (last lane of 2B and 2C). To confirm we could biotinylate full-length N171-18Q, we expressed recombinant N171-18Q in *E. coli*, purified the protein and tested whether it could be biotinylated. We also mixed the pure protein with lysates of HEK293 cells prior to biotinylation and immunoprecipitation to simulate the experiments described in Figure 3. Under these conditions, we could immunoprecipitate biotinylated full-length N171-18Q and detect it with both streptavidin and htt antibodies as both a pure protein and after mixture with HEK293 cell lysate (Fig. S2). This result suggests that the cysteines in N171-18Q protein made in living HEK293 cells may be modified in some manner that inhibits modification by the biotinylation reagent. Overall, the data indicate that the C-terminus of cp-A/1 is likely to reside C-terminal to the cysteine at residue 105. Whether cysteine 109 is also present in this fragment is unknown and we cannot be certain as to whether there is a single C-terminal residue of this fragment.

### Search for the proteolytic activities capable of cleaving htt

To determine whether the expression profile of proteases in HEK293 cells was in any way unique, we used whole genome microarray to identify the expressed genes of these cells. For comparison, we also performed an array analysis of genes expressed in mouse LTK^−^ cells (see Fig. 1). Next, we used the Gene Ontology website (http://www.geneontology.org/; GO ID, GO:0008233) and MEROPS websites (http://merops.sanger.ac.uk/; [30]) to identify the protease genes expressed in these cell lines. There are 590 proteases identified in the human genome and 592 identified in the mouse genome. Using a conservative criterion of expression above background, we found 556 and 529 protease genes showed signal intensities on arrays that met call criteria for designation as expressed in HEK293 and LTK(-) cells, respectively (see Data Files S1 and S2). Comparing the expression profiles between the two cell lines revealed 129 proteases that were specifically expressed in HEK293s (Table S2). This list of proteases was considered as possible candidates that could be responsible for cleaving httN171-18Q in HEK293 cells to a fragment that resembles cp-A. We considered the feasibility of testing each of these proteases by siRNA knock-down by testing a small subset of the list that we considered to be strong candidates based on known expression in brain, known specificity, or subcellular location. However, this approach proved to be fraught with difficulty as it became evident very quickly that we could typically only achieve about 50% knockdown of target mRNA and we were not confident that we could detect a 50% reduction in htt cleavage by the methods available (immunoblotting of transiently transfected cells). Additionally, we could not be confident as to whether a lack of activation of a protease in LTK^−^ cells accounted for the difference in ability to cleave htt N171-18Q rather than a difference in expression. Ultimately, these expression data from the HEK293 cells indicated that most of the known human protease genes are expressed in these cells.

**Figure 4:**
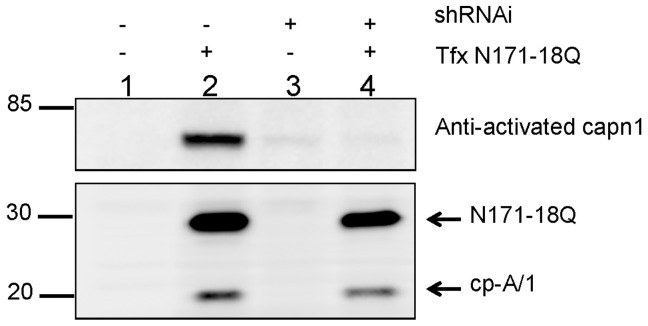
Knock-down of calpain-1 does not block htt cleavage. HEK293 cells which stably express a shRNA against calpain-1 (shRNAi +) do not block cleavage of htt following transfection with cDNA encoding N171-18Q (Tfx +). Knock-down of capn1 was confirmed by immunoblot with an antibody to activated calpain-1. Htt cp-A/1 was detected with the htt81-90 (1∶3000) antibody. The images shown are representative of at least 3 repetitions of the experiment.

We then used a bioinformatic approach, in which homology between htt and known substrates were compared, to search for proteases that may cleave in the suspected region of cp-A/1 proteolysis. We found that htt residues 85–95 have significant homology to calpain-1 (capn1) substrates; residues at positions P3, P2, and P2′ match exactly with a consensus capn1 sequence, and P1′, P3′, P5′, and P7′ rank in the top three ([31]; Fig. S3). Capn1 mRNA expression was detected in microarray analysis the 293 cells. To directly test whether capn1 generates cp-A/1, we generated HEK293 cells that stably express a shRNA against capn1. Following transfection of these capn1 knock-down cells with cDNA encoding htt N171-18Q, we found no attenuation in the production of cp-A/1 (Fig. 4). To confirm knock-down of capn1, we probed immunoblots with an antibody raised against first 7 amino acids of the activated large subunit of capn1 (μ-calpain; LGRHENA) that is generated by autolysis (a generous gift of Dr. Kevin Wang, University of Florida [26]). This antibody is similar to antibodies against the first 5 amino acids of the activated capn1 large subunit (LGRHE) generated by Saido et al [32] and independently generated by Meyer et al [33] and recognizes a neoepitope created by autolysis of the enzyme as it is activated. Transfection of naïve HEK293 cells to express N171-18Q induced activation of calpain as detected by this antibody (Fig. 4, lane 2). In cells stably expressing the shRNAi for capn1, the antibody detected little or no activated enzyme in cells transfected with N171-81Q constructs (Fig. 4, lane 4). Capn1 is activated by micromolar concentrations of Ca^++^ and it would appear that transient transfection of HEK293 cells with vectors for N171-18Q released a sufficient level of Ca^++^ to activate capn1. We did not observe a decrease in htt N171-18Q cleavage that was proportional to the decrease in activated capn1 levels in the HEK293 cells stably expressing shRNAi to capn1.

We continued to analyze htt residues for known substrate sequences as a means to identify the protease involved in generating cp-A/1. To do this analysis, we utilized the online peptidase database, MEROPS (http://merops.sanger.ac.uk/; [30]). We queried its search option for proteases by assessing possible cleavage events between two htt residues at a time, moving from the N- to C-terminus of residues 85 through 115. For example, we input htt P86 and L87 as P1 and P1′, respectively, and proceeded sequentially to the C-terminus of htt ending with Q115. We then tabulated the number of human proteases listed (Table S3). All peptide bonds except one (C109-E110) were a substrate for at least one human protease. To narrow the list of candidates, we focused on proteases that cleave near presumptive cleavage sites at I108-I112, as suggested by Lunkes *et al.* [7] (Table S4). The majority of proteases that can cleave in these regions are metallopeptidases, but we also observed representatives from cysteine, serine, and aspartyl protease families.

We next attempted to gain further information on the nature of the protease involved by incubating the transfected cells with a panel of well characterized protease inhibitors. The paradigm used involved transient transfection of the cells followed by 24 hour incubation, exchanging medium with medium containing the inhibitor, and an additional 24 hour incubation before harvest. In this HEK293 cell model of transient transfection with N171-18Q vectors, we do not observe any accumulation of cp-A/1 at 24 hours; only after 48 hours post-transfection do we detect the accumulation of a cp-A/1 fragment (not shown). Unfortunately, we were unable to identify any protease inhibitors that blocked cleavage of N171-18Q to cp-A/1 (Fig. S4). Previous studies using a panel of broad-spectrum protease inhibitors have found that the aspartyl protease inhibitor, Pepstatin A, can block generation of cp-A/1 [7], [18]. To investigate further, we repeated the experiment with a higher concentration (100 µM) of Pepstatin A, finding the same outcome of no change in cp-A/1 production (Fig. S4B). Thus, unfortunately, we were unable to narrow the candidate list generated by MEROPS database through the use of inhibitors.

**Figure 5:**
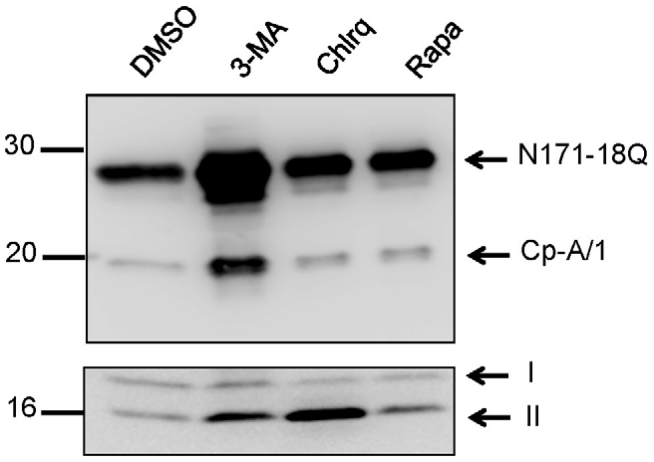
Autophagy activators or inhibitors do not block htt proteolysis. Top: inhibitors (3-methyladenine, 3-MA and Chloroquine, Chlrq) or activators (Rapamycin, Rapa) of autophagy failed to block proteolysis, as detected by immunoblot with 2B4 antibody (1∶1000). Treatment with 3-MA caused an increase in cp-B/2 and cp-A/1. Bottom: Detection of LC3-I and LC3-II. LC3 antibody was used at 1∶1000. The images shown are representative of at least 3 repetitions of the experiment.

We next examined whether the autophagy/lysosomal degradation pathways played a role in generating cp-A/1. Autophagy can coordinate with proteasomal degradation, and activation of autophagy has been shown to be protective in models of HD by enhancing removal of pathologic aggregates [reviewed in [34]]. To examine the role of autophagy in generating cp-A/1, transfected cells were treated with an inhibitor (3-methyladenine) or an activator (rapamycin) of autophagy, or chloroquine, which increases lysosomal pH and inhibits autophagic flux while still permitting autophagosome sequestration. Treating N171-18Q-transfected cells with these molecules did not block the generation of cp-A/1; 3-methyladenine (3-MA) caused an obvious increase in the accumulation of N171-18Q and cp-A/1 (Fig. 5). The effectiveness of these pharmacological agents was assessed by immunoblotting for microtubule associated protein light chain 3-I (LC3-1). Upon activation of autophagy, LC3-1 is modified by covalent attachment of phosphatidylethanolamine to generate a faster migrating LC3-II [for review see [35](Fig. 5). A small, but reproducible increase in LC-II levels was observed in cells treated with rapamycin as expected [35] (Fig. 5). Similar to what has been reported for retinal pigment epithelial cells treated with chloroquine [36], the levels of LC3-II were increased in HEK293 cells treated with this lysosomal inhibitor (Fig. 5). Counter to what would be predicted [35], the levels of LC3-II in HEK293 cells treated with 3-MA were elevated and thus, we could not confirm that 3-MA had indeed inhibited autophagy in the transfected cells.

One protease identified in our MEROPS analyses that had multiple potential cleavage sites within htt residues 86-115 was High temperature requirement A2 (HtrA2). HtrA2, once released from the mitochondrion, where it is thought to be protective in HD [37], initiates a cascade of apoptosis in the cytoplasm, preventable with use of the HtrA2 inhibitor Ucf-101 [38]. In a cellular model of HD, treatment with Ucf-101 reversed the apoptotic signal by blocking the degradation of Inhibitor of Apoptosis Protein-1 [39]. To test whether HtrA2 may also cleave htt, we incubated increasing concentrations of Ucf-101 with N171-18Q transfected HEK293 cells. We found that Ucf-101 failed to abrogate production of cp-A/1 suggesting HtrA2 does not cleave htt N171-18Q (Fig. S5).

**Figure 6:**
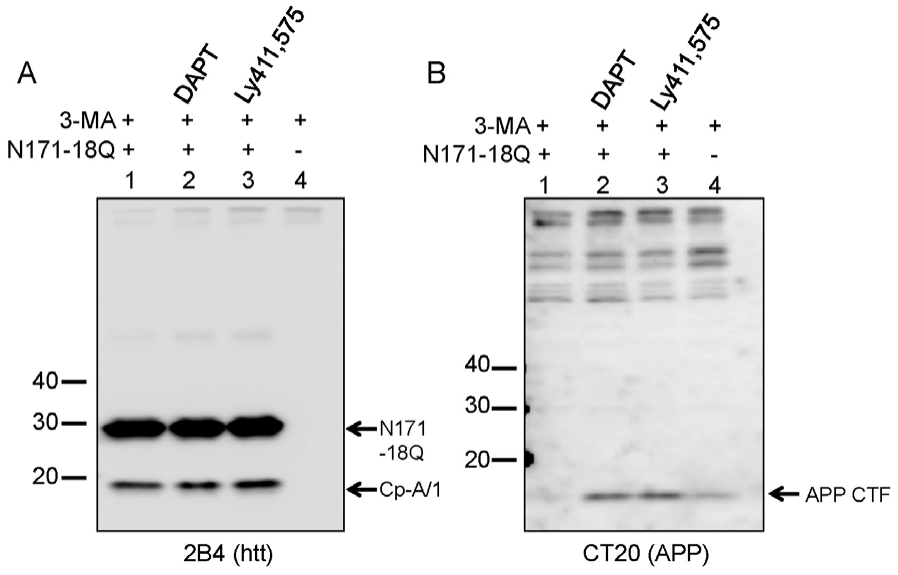
Gamma-secretase inhibitors fail to block htt proteolysis. A, HEK293 cells were transfected with N171-18Q, treated with 3-MA, and either untreated, or treated with DAPT or LY411,575 followed by immunoblot analysis for htt (lane 4 is non-transfected). In all treatment scenarios, no change was detected in htt proteolysis; cp-A/1 (arrowhead on left panel) was detected at equivalent levels regardless of treatment. B, The efficacy of the γ-secretase inhibitor was confirmed by immunoblotting for APP C-terminal fragments (10 kDa CTF; arrow on right panel). The images shown are representative of at least 3 repetitions of the experiment.

The next class of protease inhibitors we interrogated more closely was inhibitors of γ-secretase. A recent study by Kegel *et al.* reported that inhibitors of the enzyme complex termed γ-secretase, which is involved in the intramembranous cleavage of multiple substrates including the amyloid precursor protein (APP), reduce the generation of cp-A/1 [40]. Using our model of HEK293 cell transient transfection, we tested two compounds, termed DAPT and LY411,575, that have been shown to have good specificity for inhibiting γ-secretase [41], [42]. In these experiments, we included 3-MA as a means to slow degradation of N171-18Q and enhance the accumulation of cp-A/1 fragments. Both of the γ-secretase inhibiting compounds proved effective in elevating the levels of C-terminal fragments of the endogenous amyloid precursor protein (Fig. 6), which occurs upon inhibiting γ-secretase [43]. However, neither compound had any obvious effect on generation of cp-A/1 by cleavage of htt N171-18Q fragments (Fig. 6). Thus, we cannot confirm a role for γ-secretase in the generation of htt cp-A/1 fragments in our cell model of htt cleavage.

A last protease candidate we examined was insulin degrading enzyme (IDE), which is an intracellular protease [for review see [44]] that has been shown to cleave multiple substrates [45], [46]. IDE can be inhibited by excess insulin [45] and we tested whether IDE might be involved in the cleavage of htt by incubating transiently transfected HEK293 cells in high levels of insulin (10 µM = 58 µg/ml). No obvious effect on the generation of cp-A/1 was observed (Fig. S6).

## Discussion

This study utilized a previously reported cell culture model of htt proteolysis to further characterize the generation of the predominant htt fragment in human HD pathology termed cp-A/1. The HEK 293 cell model could be a useful platform to identify proteases capable of generating cp-A/1. Our results indicate that, in these cells, htt fragments produced by calpain, caspase, or MMP-10 cleavage are rather weak substrates for the production of cp-A/1 and cp-B/2; whereas fragments of cp-B/2 size are a preferred substrate. Using cysteine labeling reagents, we demonstrate that we can detect a cp-A/1 fragment produced in HEK293 cells that possess at least one cysteine of which the only candidates are located at position 105 and 109. This result places the cleavage site very close to what has previously been reported by Lunkes *et al.* [7] and Kim *et al* [18]. Using shRNAi technology, we show that lowering the levels of activated calpain does not produce a proportional reduction in the generation of cp-A/1. We also find no evidence to support the idea that γ-secretase directly generates cp-A/1 from cp-B/2 sized fragments. Unfortunately, we were unable to identify a protease inhibitor that showed an appreciable efficacy in reducing the conversion of cp-B/2 sized fragments to cp-A/1 sized fragments. The most robust effect observed was a dramatic increase in the levels of cp-B/2 and cp-A/1 in cells treated with 3-methyladenine. Collectively, our results are consistent with the hypothesis that HEK293 cells express a novel protease that cleaves htt between amino acids 105 and 114 to produce an htt fragment that possesses antibody-binding properties that are identical to the primary pathologic fragment that produces inclusions in HD brain [8].

### Substrates for the generation of cp-A/1

A recent study identified fourteen htt cleavage products in the brains of *Hdh*Q150 knock-in mice [9]. Eight of these cleavage products appear to be similar to fragments that have been described elsewhere: cp-A/1 and cp-B/2 [7], [20], an MMP-10 site at 402 [24], two calpain sites at residues 469 and 536 [23], two caspase-2/3 sites at 513 and 552 [22], and a caspase-6 site at 586 [47]. It was unclear whether these fragments, cp-B/2 and longer, can be cleaved to cp-A/1. In a recent study of transgenic mice that express htt cDNA engineered to mimic the caspase 6 fragment (N586), we demonstrated both N586-82Q and N586-18Q can be cleaved *in vivo* to produce cp-A/1 sized fragments [48]. Thus, it is clear that full-length htt can be processed to shorter htt fragments and that at least some htt N-terminal fragments can be further cleaved to produce cp-A/1 sized fragments.

In the HEK293 cell model, we find that cp-B/2 sized fragments seem to be very efficiently cleaved to produce cp-A/1 sized fragments whereas N-terminal fragments corresponding to natural products of calpain, caspase, and metalloproteinases were rather inefficiently cleaved. It is notable that we were unable to detect endogenous cp-B/2 or cp-A/1 fragments in these HEK293 cells (Fig. S7), which express endogenous human htt, suggesting that either some intermediate exists between full-length htt and cp-B/2 and cp-A/1, or that these cleavage products are produced at such a low level that they are not detectable unless precursor substrates are over-expressed. It is also noteworthy that we observed rather limited accumulation of cp-B/2 sized fragments in HEK293 cells expressing htt N586 fragments. It is possible that htt must first be cleaved to cp-B/2 and that HEK293 cells do not express high levels of the protease responsible for generating cp-B/2 or the expressed protease is not activated. It is also possible that htt fragments that are longer than any we tested are better substrates to generate cp-B/2 and that there is a preferred intermediate fragment that we have yet to identify. Overall, our data indicate that the protease in HEK293 cells that is capable of producing a cp-A/1 sized fragment prefers a cp-B/2 sized htt substrate.

### Proteolytic inhibitors in HEK293 cells

The precise identity of the protease(s) responsible for the generation of cp-A/1-like fragments in these HEK293 cells, or any other system, remains unknown. In each attempt to identify an inhibitor of cp-A/1 production we were unsuccessful. HEK293 cells that stably express shRNAi for calpain 1 showed much lower levels of activated calpain 1, which was induced by transfection, than control cells. Thus, it seems unlikely that calpain-1 is responsible for generating cp-A/1 in our HEK293 cell model. Additionally, we found that the calpain inhibitor MDL28170, which inhibits both capn1 and capn2, failed to block the generation of cp-A/1. Finally, expression analyses indicated that capn1 and capn2 mRNA were expressed in the mouse LTK- cells that show no production of cp-A/1 (see Data File S2). Together, these data indicate that calpain cleavage is unlikely to be involved in generating cp-A/1 in the HEK 293 cells.

In an effort to narrow the focus in the search for cp-A/1 generating proteases, we tested a panel of chemical inhibitors. Unfortunately, none of the inhibitors were efficacious in reducing the generation of cp-A/1, but some of the outcomes were more definitive than others. First, we have high confidence that γ-secretase inhibitors DAPT and Ly411,575 do not lower the generation of cp-A/1. For these inhibitors, we were able to demonstrate efficacy by showing an accumulation of APP C-terminal fragments, which occurs when γ-secretase is inactive [43]. We also have high confidence that the proteasome and lysosomal proteases are not involved as we observed obvious evidence that these compounds were active within the cell. The evidence included enhanced accumulation of cp-B/2 and cp-A/1 in the case of the proteasome inhibitor MG132, and enhanced accumulation of LC3-II in cells treated with chloroquine. This latter effect has been previously reported in retinal epithelial cells [36]. Thus, we have high confidence in concluding that the proteasome or lysosomal systems are not directly involved in generating cp-A/1. However, these systems are involved indirectly because they appear to govern the stability of cp-B/2 substrate.

For the remaining panel of inhibitors, we have little corroborating evidence that the inhibitor entered cells and engaged the target proteases. It is difficult to produce positive controls for each of these inhibitors and thus the absence of efficacy produces a negative outcome that is limited in interpretation. One interpretation is that the protease responsible for generating cp-A/1 acts via a novel mechanism and is uniquely resistant to these inhibitors. Another interpretation is that for some reason the HEK293 cells were impermeable to the inhibitor that would target cp-A/1 generating protease. We were particularly surprised by the inability of Pepstatin A to inhibit the generation of cp-A/1 because previous studies have shown efficacy with this inhibitor [7], [18]. Whether our findings are due to differences in the cell types used (NG108 and X57 cell lines versus HEK293 cells) is yet to be resolved. Thus, we essentially reach an impasse in which the inhibitor studies in the HEK293 cells were uninformative regarding the identity of the protease.

### Autophagy inhibitors and htt fragment stability

One of the most striking effects we observed was the dramatic accumulation of both the precursor htt N171-18Q and cp-A/1 cleavage product in cells treated with 3-methyladenine. This compound is described as a potent inhibitor of autophagy [35] and thus we attribute the accumulation of these htt fragments in the presence of 3-methyladenine to a blockade of autophagy. However, as noted in Figure 5, cells treated with this compound did not show the expected accumulation of LC3-I and thus we cannot reliably conclude that the accumulation of htt fragments in the presence of this compound is due to an inhibition of autophagy. 3-methyladenine has been shown to inhibit other cellular processes [for review see [35]] and it is possible that the accumulation of htt fragments is due to an effect of the compound on a process other than autophagy. However, we did observe that rapamycin treatment induced the expected modification of LC3 that is associated with activation of autophagy and yet we observed no increase in cp-A/1 production. This observation suggests that autophagy is probably not involved in generating cp-A/1 in the HEK 293 cells.

## Conclusions

In summary, we have determined that cp-B/2-like htt fragments are efficiently cleaved substrate to produce cp-A/1 in HEK293 cells. We also provide evidence that in this cell model, cp-A/1 is generated by a cleavage event C-terminal to the cysteine residue at 105; a site similar to that identified by Lunkes *et al.* [7]. Lacking precise information, we cannot rule out the possibility that there are multiple C-termini for cp-A/1.

Although it is possible the protease in human HEK293 cells that cleaves cp-B/2 to cp-A/1 is not the same protease that is responsible for generating the cp-A/1 sized pathologic fragment in human HD brains, the antibody binding characteristics of the cp-A/1 fragment found in HEK293 cells is very similar to that of the pathologic fragment in inclusions [7], [8]. From a combination of genetic and pharmacological inhibition of calpain-1, HtrA2, and γ-secretase, we are fairly confident that cleavage of cp-B/2 to cp-A/1 in the HEK293 cell model does not involve these proteases. Notably LY411,575 has also been reported to inhibit signal peptide peptidase [49], and thus this protease is also unlikely to be involved. We are similarly confident that cleavage of cp-B/2 to cp-A/1 does not involve the proteasome or lysosomal proteases. Our data are consistent with the notion that human cells produce a novel protease that cleaves cp-B/2 sized fragments of htt to create pathologically relevant cp-A/1 sized fragments.

## Supporting Information

Table S1 The sequences of sense (S) and antisense primers (AS) that were used to generate some of the htt cDNAs described in this study.
(DOC)

Table S2 List of protease genes in which mRNA expression was specifically detected in the HEK293 cells as compared to the mouse L cells.
(XLS)

Table S3 The number different human proteases that could cleave htt between residues 86–115 is shown. The left column shows the amino acid position of htt (based on 23Q; GenBank NM_002111). The second column shows the substrate sequence for htt amino acids that are at the designated amino acid positions. The last column shows how many human proteases are reported to cleave the bond shown in the second row. Residues 98–99 are not shown because that sequence is K-K, which is the same as residues 91–92. No human proteases were found that could cleave between htt amino acid sequences 109–110.
(DOCX)

Table S4 The names of human proteases that may cleave at potential cleavage sites in specific areas of htt between residues 108–115 are listed. The first column lists the htt amino acid position, followed by the specific residue sequence able to be cleaved. The last column lists the name of the protease with the MEROPS identification number in parentheses. Most of the proteases listed are extracellular-activated matrix metalloproteinases. HtrA2 is a cytosolic protease previously implicated in HD (see Results).
(DOCX)

Figure S1 **Recombinant N-terminal fragments of htt corresponding to natural cleavage products of calpain, caspase, and MMP-10 are inefficiently cleaved to cp-A/1 and cp-B/2.** Vectors engineered to express N-terminal htt fragments (18Q) were transfected into HEK293s, treated with 3-MA after 24 hours (to stabilize cleavage products – see Figure 5 of main text), and then after another 24 hours cell lysates were analyzed by immunoblot (NTf, non-transfected cells). A, This membrane was incubated with EM48 at 1∶500, and shows the relative amounts of cp-A/1 from each substrate. B, The same cell lysates were analyzed separately using the antibody 1H6 at 1∶2000 to confirm cleavage fragment identity. The images shown are representative of at least 3 repetitions of the experiment. The positions of the bands that appear to be cp-A/1 and cp-B/2 are marked by arrows. The arrowhead marks the position of a band that migrates to a position expected for a dimer, which is present to variable levels in transiently transfected cells.
(TIF)

Figure S2 **Full-length, purified N171-18Q can be biotinylated.** Recombinant N171-18Q was incubated in the presence (+) or absence (−) of cell lysate and/or biotinylation reagent, followed by immunoprecipitation (IP) with the antibody htt64-82. Separate membranes were then incubated with HRP-conjugated streptavidin (A) or htt3-16 (B) antibodies to confirm the presence of htt. N171-18Q (bottom arrow, cp-B/2) was seen to homodimerize (*) and this dimer was of similar size to IgG used in the IP (upper arrow, IgG). The images shown are representative of at least 2 repetitions of the experiment.
(TIF)

Figure S3 **Htt residues 85–95 have homology to calpain-1 substrates.** The positions of a putative substrate are shown at the top row, followed by an alignment of the preferred substrate for calpain-1 (capn1) and the htt sequence. Htt residues in bold are top matches to the preferred capn1 substrate at that position, capital letters rank in the top three, and lower case are outside of the top three.
(TIF)

Figure S4 **Broad-spectrum protease inhibitors fail to block htt cleavage.** A, Representative immunoblots of HEK293 cells transfected with htt and treated with various protease inhibitors (see Methods for concentrations) for 24 hours show that no class of inhibitors was able to block proteolysis. 0.1% DMSO served as a control. EM48 was used at 1∶500 to detect htt. B, A higher concentration of Pepstatin A, 100 µM, was used to compare to previous studies; 1% DMSO served as a control. NT is cells not treated with any molecule or diluents. The antibody used was htt1-17 (1∶6000). The images shown are representative of at least 3 repetitions of the experiment.
(TIF)

Figure S5 **An HtrA2/Omi inhibitor does not block htt cleavage.** Htt-transfected cells were either non-treated (NT) or treated with the HtrA2 inhibitor Ucf-101, followed by lysis and analysis by immunoblot. DMSO at 0.1% was a control. No detectable decrease in cleavage was observed over a range of inhibitor concentrations, as observed with the EM48 antibody (1∶500). The images shown are representative of at least 3 repetitions of the experiment.
(TIF)

Figure S6 **Incubation of HEK293 cells in medium with high levels of insulin does not diminish the cleavage of htt N171-18Q to cp-A/1.** HEK293 cells were transfected with vectors for htt N171-18Q as described in Methods. 24 hours after transfection, insulin was added to the medium to a final concentration of 10 µm and the cells were incubated a further 24 hours before harvest and immunoblot analysis with the antibody 2B4. For these experiments, cells were lysed by freeze thaw in PBS (2 cycles dry ice/ethanol bath and 42°C water bath) with insoluble material removed by centrifugation at 3000×g for 2 minutes. The image shown is representative of at least 3 independent repetitions of the experiment.
(TIF)

Figure S7 **Lack of accumulation of cp-A/1 like fragments in untransfected HEK293 cells.** We compared untransfected HEK293 cells to cells transiently transfected with httN171-18Q for 48 h. Cells were treated with MG132 (lane 2 and 5) or chloroquine (lanes 3 and 6) to stabilize any htt fragments that may have been produced. We observe no obvious endogenous htt cleavage products in the HEK293 cells.
(TIF)

Data File S1 **Excel spreadsheets of raw data for expression profile of the HEK293 cells. See** **Methods** **for an explanation of how the data were analyzed.**
(XLS)

Data File S2 **Excel spreadsheets of raw data for expression profile of the mouse L-TK- cells. See** **Methods** **for an explanation of how the data were analyzed.**
(XLS)
