# Supplementary material for: Analysis of Proteolytic Processes and Enzymatic Activities in the Generation of Huntingtin N-Terminal Fragments in an HEK293 Cell Model
Source: PLoS One. 2012 Dec 7;7(12):e50750. doi: 10.1371/journal.pone.0050750 (PMC3517621; doi:10.1371/journal.pone.0050750)
Supplement: Table S1 — The sequences of sense (S) and antisense primers (AS) that were used to generate some of the htt cDNAs described in this study. (DOC) [file pone.0050750.s001.doc]

**Supplementary Table S1**. The following sense (S) and antisense primers (AS) were used to generate some of the cDNAs used for cloning

| Primer name | Sequence |
| --- | --- |
| Htt-171-S | 5’- GCTTACAGCTCGAGCCTCTATA - 3’ |
| Htt-402-STOP-AS | 5’- GGCCTCGAGTTAGCCCCCGACTGCGGT - 3' |
| Htt-469-STOP-AS | 5’- GGCCTCGAGTTATGTTAAGGCAGAGCT - 3’ |
| Htt-513-STOP-AS | 5’- GGCCTCGAGTTAATCCACTGAGTCCGC - 3’ |
| Htt-536-STOP-AS | 5’- GGCCTCGAGTTAGGAGCTGTGGCTCAA - 3’ |
| Htt-552-STOP-AS | 5’- GGCCTCGAGTTAATCATTCAGGCTCAT - 3’ |
