# Supplementary material for: Analysis of Proteolytic Processes and Enzymatic Activities in the Generation of Huntingtin N-Terminal Fragments in an HEK293 Cell Model
Source: PLoS One. 2012 Dec 7;7(12):e50750. doi: 10.1371/journal.pone.0050750 (PMC3517621; doi:10.1371/journal.pone.0050750)
Supplement: Table S3 — The number different human proteases that could cleave htt between residues 86–115 is shown. The left column shows the amino acid position of htt (based on 23Q; GenBank NM_002111). The second column shows the substrate sequence for htt amino acids that are at the designated amino acid positions. The last column shows how many human proteases are reported to cleave the bond shown in the second row. Residues 98–99 are not shown because that sequence is K-K, which is the same as residues 91–92. No human proteases were found that could cleave between htt amino acid sequences 109–110. (DOCX) [file pone.0050750.s003.docx]

**Supplemental Table S3.** The number different human proteases could be able to cleave htt between residues 86-115 is shown. The left column shows the amino acid position of htt (based on 23Q; GenBank NM_002111). The second column shows the substrate sequence for htt amino acids that are at the designated amino acid positions. The last column shows how many human proteases are reported to cleave the bond shown in the second row. Residues 98-99 are not shown because that sequence is K-K, which is the same as residues 91-92. No human proteases were found that could cleave between htt amino acid sequences 109-110.

Supplemental Table S3. Number of human proteases that cleave htt residues 86-115.

| Htt amino acid position | Substrate sequence | Number of proteases |
| --- | --- | --- |
| 86-87 | P-L | 12 |
| 87-88 | L-H | 6 |
| 88-89 | H-R | 4 |
| 89-90 | R-P | 6 |
| 90-91 | P-K | 5 |
| 91-92 | K-K | 15 |
| 92-93 | K-E | 6 |
| 93-94 | E-L | 17 |
| 94-95 | L-S | 16 |
| 95-96 | S-A | 12 |
| 96-97 | A-T | 13 |
| 97-98 | T-K | 3 |
| 99-100 | K-D | 6 |
| 100-101 | D-R | 15 |
| 101-102 | R-V | 25 |
| 102-103 | V-N | 4 |
| 103-104 | N-H | 3 |
| 104-105 | H-C | 1 |
| 105-106 | C-L | 5 |
| 106-107 | L-T | 16 |
| 107-108 | T-I | 10 |
| 108-109 | I-C | 2 |
| 109-110 | C-E | 0 |
| 110-111 | E-N | 7 |
| 111-112 | N-I | 10 |
| 112-113 | I-V | 3 |
| 113-114 | V-A | 5 |
| 114-115 | A-Q | 12 |
