# Supplementary material for: Analysis of Proteolytic Processes and Enzymatic Activities in the Generation of Huntingtin N-Terminal Fragments in an HEK293 Cell Model
Source: PLoS One. 2012 Dec 7;7(12):e50750. doi: 10.1371/journal.pone.0050750 (PMC3517621; doi:10.1371/journal.pone.0050750)
Supplement: Table S4 — The names of human proteases that may cleave at potential cleavage sites in specific areas of htt between residues 108–115 are listed. The first column lists the htt amino acid position, followed by the specific residue sequence able to be cleaved. The last column lists the name of the protease with the MEROPS identification number in parentheses. Most of the proteases listed are extracellular-activated matrix metalloproteinases. HtrA2 is a cytosolic protease previously implicated in HD (see Results). (DOCX) [file pone.0050750.s004.docx]

**Supplemental Table S4.** The names of human proteases that may cleave at potential cleavage sites in specific areas of htt between residues 108-115 are listed. The first column lists the htt amino acid position, followed by the specific residue sequence able to be cleaved. The last column lists the name of the protease with the MEROPS identification number in parentheses. Most of the proteases listed are extracellular-activated matrix metalloproteinases. HtrA2 is a cytosolic protease previously implicated in HD (see Results).

Supplemental Table 4. Human proteases are listed that have potential cleavage sites in htt at regions of interest.

| Htt amino acid position | Substrate sequence | Human proteases (MEROPS ID) |
| --- | --- | --- |
| 108-109 | I-C | Acid ceramidase precursor (C89.001)  Matrix metallopeptidase-2 (M10.003) |
| 109-110 | C-E | None |
| 110-111 | E-N | Cathepsin K (C01.036)  Matrix metallopeptidase-2 (M10.003)  Matrix metallopeptidase-3 (M10.005)  Matrix metallopeptidase-8 (M10.002)  Matrix metallopeptidase-9 (M10.004)  Membrane-type matrix metallopeptidase-1 (M10.014)  Membrane-type matrix metallopeptidase-4 (M10.017) |
| 111-112 | N-I | Cathepsin D (A01.009)  Legumain (C13.004)  Matrix metallopeptidase-1 (M10.001)  Matrix metallopeptidase-2 (M10.003)  Matrix metallopeptidase-3 (M10.005)  Matrix metallopeptidase-8 (M10.002)  Matrix metallopeptidase-9 (M10.004)  Membrane-type matrix metallopeptidase-1 (M10.014)  Pepsin A (A01.001)  Signal peptidase complex 21kD (S26.010) |
| 112-113 | I-V | Cathepsin K (C01.036)  Elastase-2 (S01.131)  Matrix metallopeptidase-9 (M10.004) |
| 87-88, 94-95, 102-103, 106-107, 107-108, 113-114 | L-H, L-S, V-N, L-T, T-I, V-A | HtrA2 (S01.278) |
