# Supplementary figures and images for: Analysis of Proteolytic Processes and Enzymatic Activities in the Generation of Huntingtin N-Terminal Fragments in an HEK293 Cell Model
Source: PLoS One. 2012 Dec 7;7(12):e50750. doi: 10.1371/journal.pone.0050750 (PMC3517621; doi:10.1371/journal.pone.0050750)

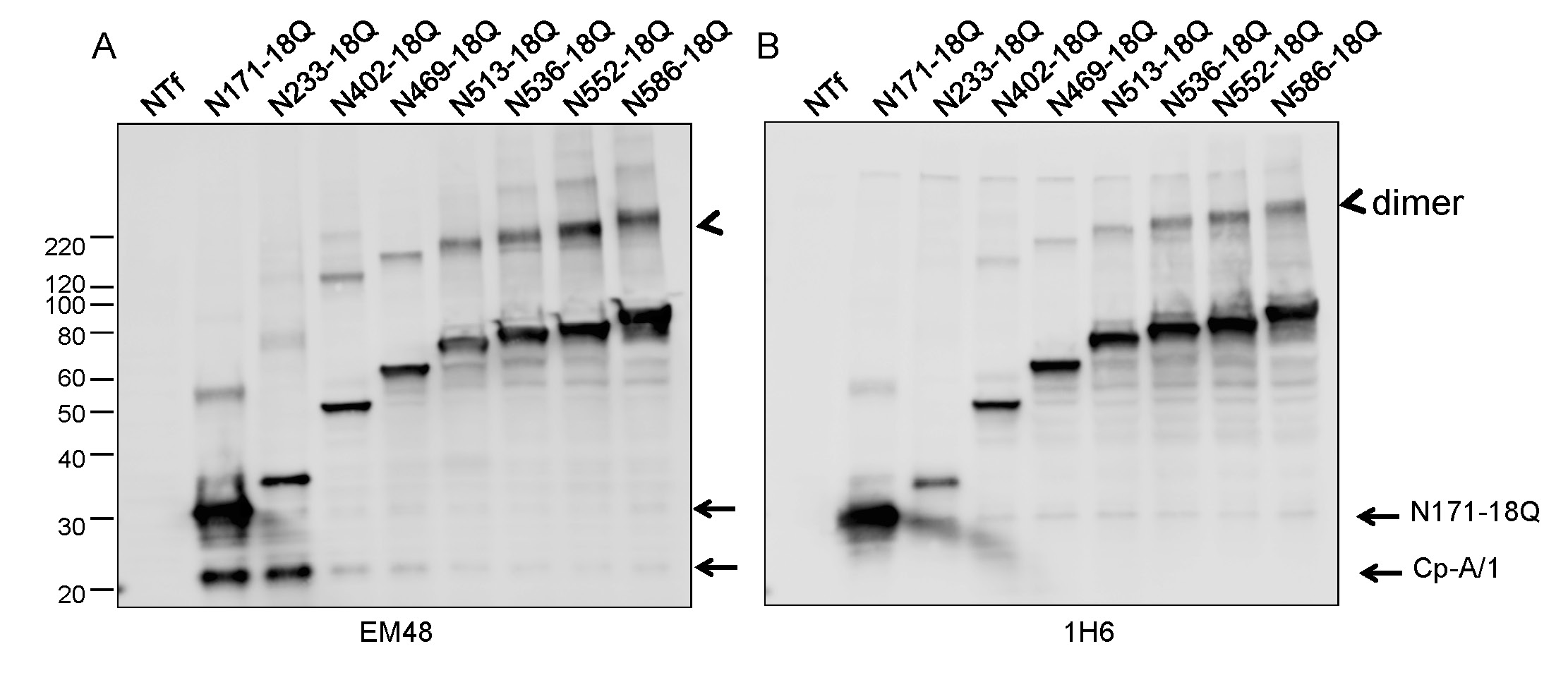

Supplement: Figure S1 — Recombinant N-terminal fragments of htt corresponding to natural cleavage products of calpain, caspase, and MMP-10 are inefficiently cleaved to cp-A/1 and cp-B/2. Vectors engineered to express N-terminal htt fragments (18Q) were transfected into HEK293s, treated with 3-MA after 24 hours (to stabilize cleavage products – see Figure 5 of main text), and then after another 24 hours cell lysates were analyzed by immunoblot (NTf, non-transfected cells). A, This membrane was incubated with EM48 at 1∶500, and shows the relative amounts of cp-A/1 from each substrate. B, The same cell lysates were analyzed separately using the antibody 1H6 at 1∶2000 to confirm cleavage fragment identity. The images shown are representative of at least 3 repetitions of the experiment. The positions of the bands that appear to be cp-A/1 and cp-B/2 are marked by arrows. The arrowhead marks the position of a band that migrates to a position expected for a dimer, which is present to variable levels in transiently transfected cells. (TIF) [file pone.0050750.s005.tif]

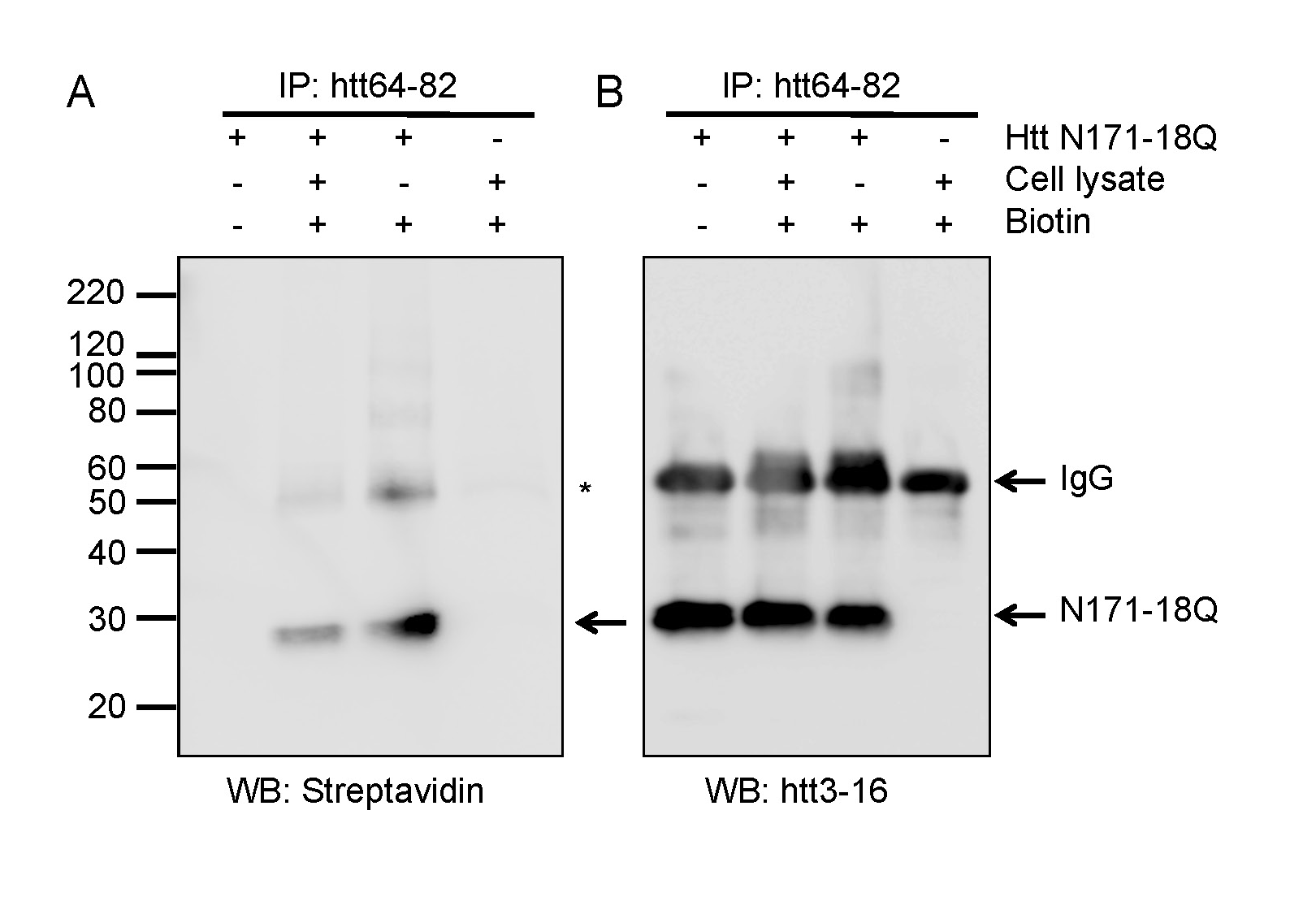

Supplement: Figure S2 — Full-length, purified N171-18Q can be biotinylated. Recombinant N171-18Q was incubated in the presence (+) or absence (−) of cell lysate and/or biotinylation reagent, followed by immunoprecipitation (IP) with the antibody htt64-82. Separate membranes were then incubated with HRP-conjugated streptavidin (A) or htt3-16 (B) antibodies to confirm the presence of htt. N171-18Q (bottom arrow, cp-B/2) was seen to homodimerize (*) and this dimer was of similar size to IgG used in the IP (upper arrow, IgG). The images shown are representative of at least 2 repetitions of the experiment. (TIF) [file pone.0050750.s006.tif]

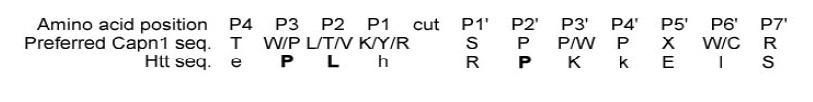

Supplement: Figure S3 — Htt residues 85–95 have homology to calpain-1 substrates. The positions of a putative substrate are shown at the top row, followed by an alignment of the preferred substrate for calpain-1 (capn1) and the htt sequence. Htt residues in bold are top matches to the preferred capn1 substrate at that position, capital letters rank in the top three, and lower case are outside of the top three. (TIF) [file pone.0050750.s007.tif]

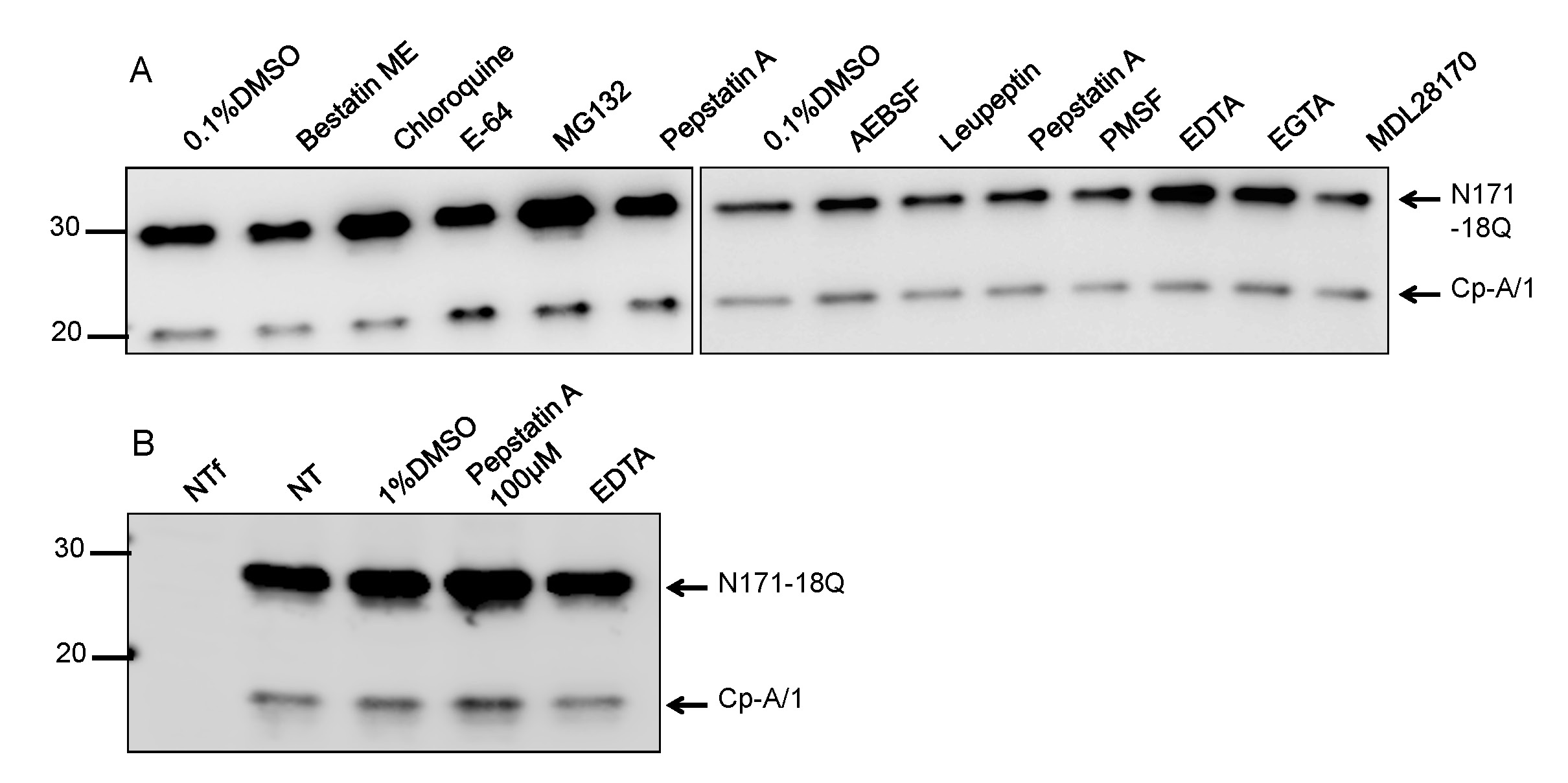

Supplement: Figure S4 — Broad-spectrum protease inhibitors fail to block htt cleavage. A, Representative immunoblots of HEK293 cells transfected with htt and treated with various protease inhibitors (see Methods for concentrations) for 24 hours show that no class of inhibitors was able to block proteolysis. 0.1% DMSO served as a control. EM48 was used at 1∶500 to detect htt. B, A higher concentration of Pepstatin A, 100 µM, was used to compare to previous studies; 1% DMSO served as a control. NT is cells not treated with any molecule or diluents. The antibody used was htt1-17 (1∶6000). The images shown are representative of at least 3 repetitions of the experiment. (TIF) [file pone.0050750.s008.tif]

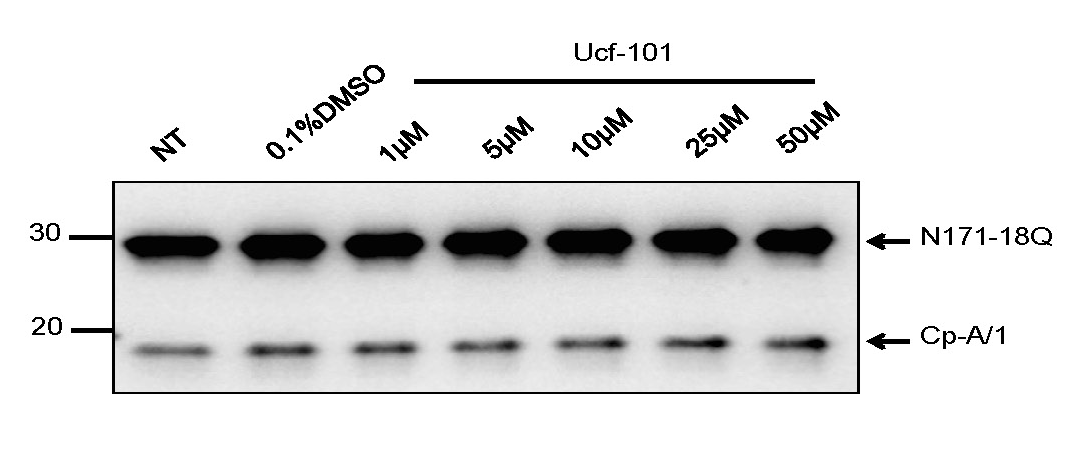

Supplement: Figure S5 — An HtrA2/Omi inhibitor does not block htt cleavage. Htt-transfected cells were either non-treated (NT) or treated with the HtrA2 inhibitor Ucf-101, followed by lysis and analysis by immunoblot. DMSO at 0.1% was a control. No detectable decrease in cleavage was observed over a range of inhibitor concentrations, as observed with the EM48 antibody (1∶500). The images shown are representative of at least 3 repetitions of the experiment. (TIF) [file pone.0050750.s009.tif]

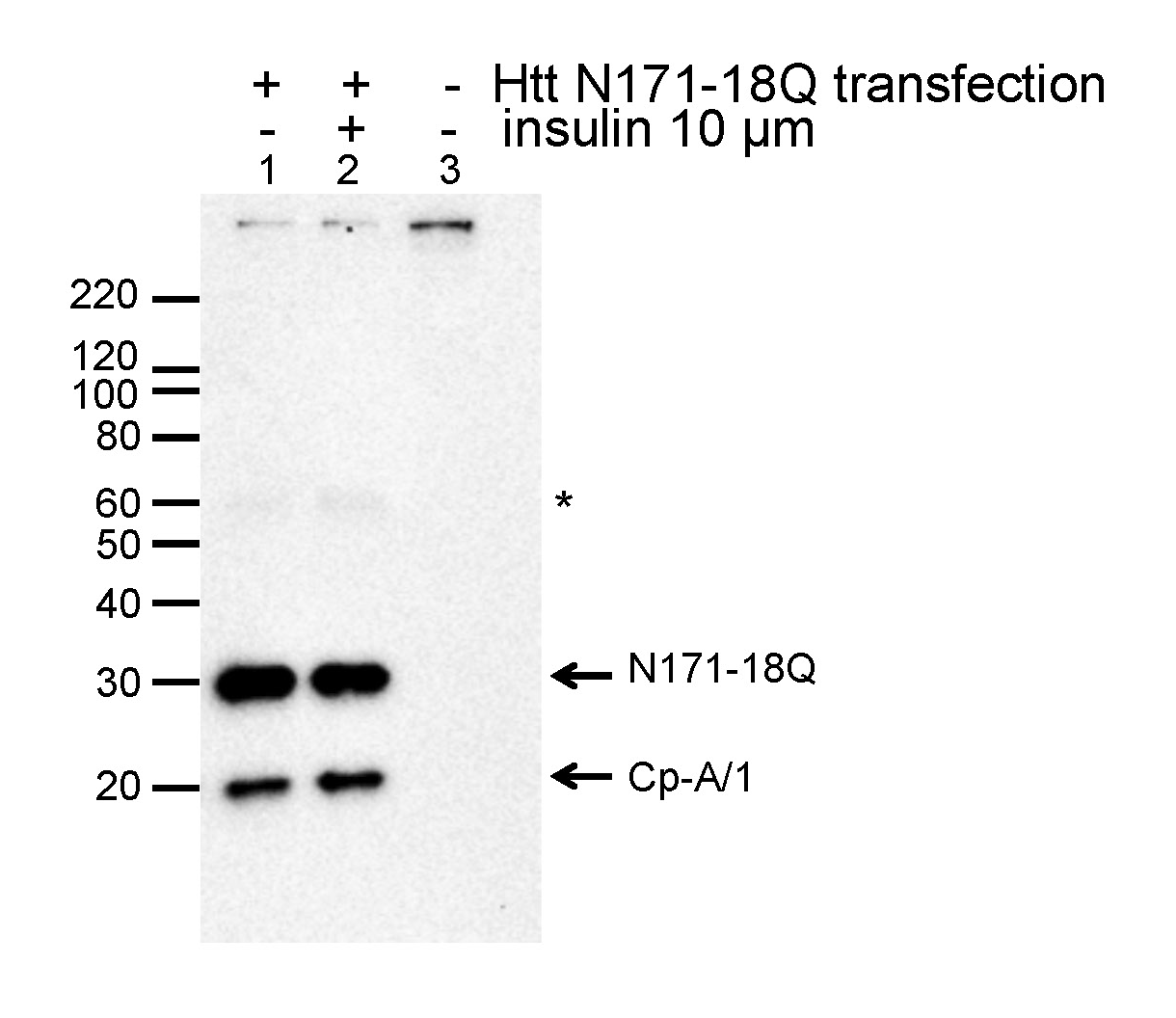

Supplement: Figure S6 — Incubation of HEK293 cells in medium with high levels of insulin does not diminish the cleavage of htt N171-18Q to cp-A/1. HEK293 cells were transfected with vectors for htt N171-18Q as described in Methods. 24 hours after transfection, insulin was added to the medium to a final concentration of 10 µm and the cells were incubated a further 24 hours before harvest and immunoblot analysis with the antibody 2B4. For these experiments, cells were lysed by freeze thaw in PBS (2 cycles dry ice/ethanol bath and 42°C water bath) with insoluble material removed by centrifugation at 3000×g for 2 minutes. The image shown is representative of at least 3 independent repetitions of the experiment. (TIF) [file pone.0050750.s010.tif]

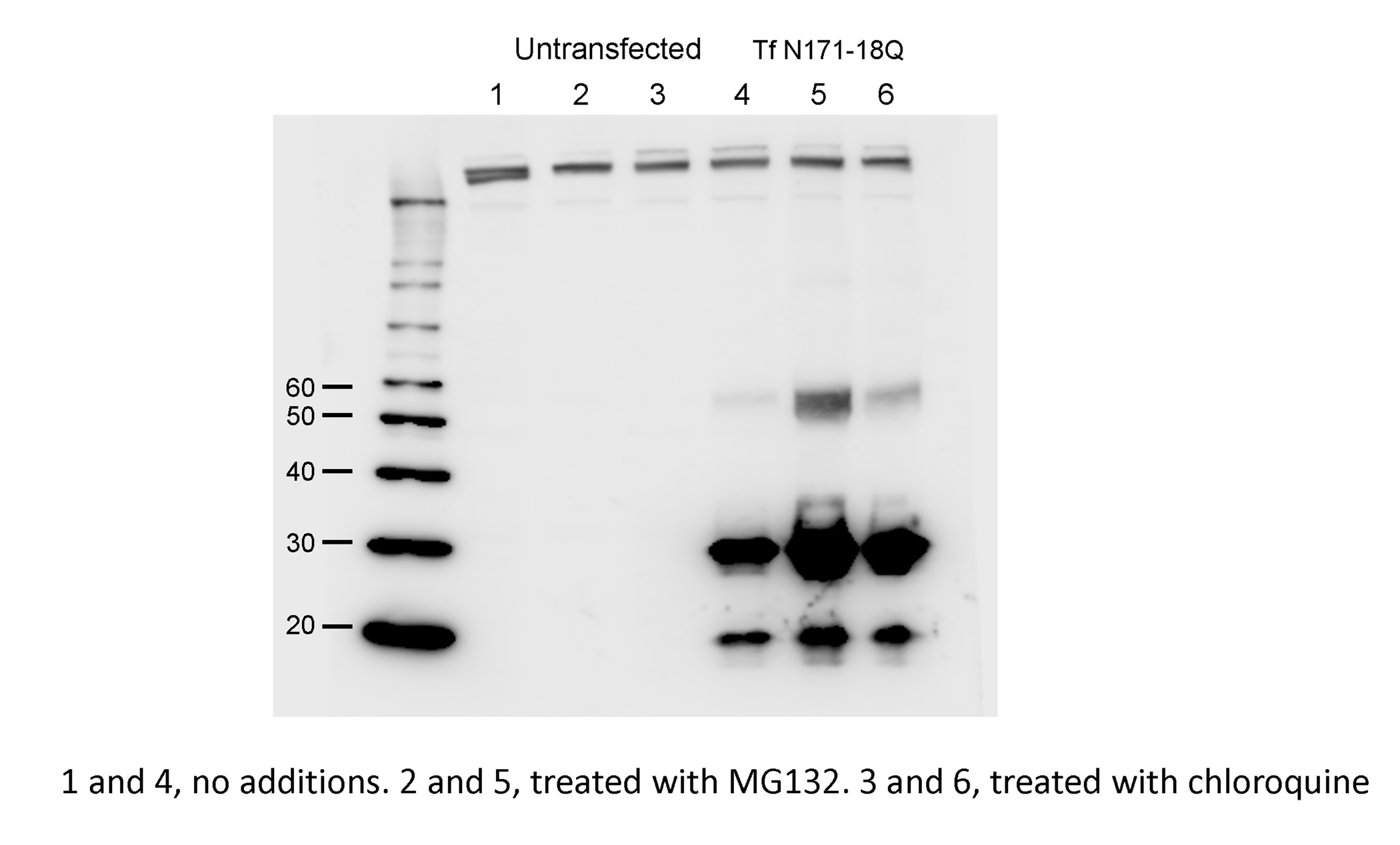

Supplement: Figure S7 — Lack of accumulation of cp-A/1 like fragments in untransfected HEK293 cells. We compared untransfected HEK293 cells to cells transiently transfected with httN171-18Q for 48 h. Cells were treated with MG132 (lane 2 and 5) or chloroquine (lanes 3 and 6) to stabilize any htt fragments that may have been produced. We observe no obvious endogenous htt cleavage products in the HEK293 cells. (TIF) [file pone.0050750.s011.tif]
